# Mycoremediation of Petroleum-Contaminated Soil Using Native *Ganoderma* and *Trametes* Strains from the Ecuadorian Amazon

**DOI:** 10.3390/jof11090651

**Published:** 2025-09-02

**Authors:** Isabel Cipriani-Avila, Cony Decock, Aracely Zambrano-Romero, Katherine Zaldumbide, Mónica Garcés-Ruiz, Jazel Caiza-Olmedo, Ana Gordillo, Verónica Luna, Patrick A. Gerin

**Affiliations:** 1 Laboratory of Bioengineering and Biorefining, Earth and Life Institute—Applied Microbiology, Université Catholique de Louvain, Croix du Sud, 2 box L7.05.19, B-1348 Louvain-la-Neuve, Belgium; 2 Grupo de Investigación en Ecología Microbiana y Microbiología Aplicada, Facultad de Ciencias Exactas, Naturales y Ambientales, Pontificia Universidad Católica del Ecuador, Quito 170143, Ecuador; 3 Mycothèque de l’Université Catholique de Louvain (MUCL, BCCM), Earth and Life Institute—Applied Microbiology, Université Catholique de Louvain, Croix du Sud, 2 box L7.05.06, B-1348 Louvain-la-Neuve, Belgium; 4 Instituto de Microbiología, Universidad San Francisco de Quito USFQ, Campus Cumbayá, Diego de Robles y Vía Interoceánica, Quito 170901, Ecuador

**Keywords:** white rot fungi, ascomycetes, basidiomycetes, bioremediation, petroleum hydrocarbons

## Abstract

Petroleum-contaminated soils are a major environmental concern worldwide. In Ecuador, extensive oil spills in the Amazon have led to widespread hydrocarbon pollution, threatening ecosystems and posing health risks to nearby communities. Conventional remediation techniques are resource-intensive and may render soil unsuitable for future use. In contrast, mycoremediation—using fungi to degrade toxic contaminants—offers a sustainable alternative. White-rot fungi, known for their ligninolytic enzyme systems such as laccases and peroxidases, are capable of degrading a wide range of organic pollutants, including petroleum hydrocarbons. This study assessed the enzymatic activity of 16 fungal strains from the phyla Ascomycota and Basidiomycota isolated in the Ecuadorian Amazon. Plate-based screening and quantitative laccase activity assays confirmed positive enzymatic activity in all strains. The five strains with the highest enzymatic activity were *Ganoderma* cf. *parvulum* QCAM7791, *Trametes menziesii* QCAM7783, *Trametes menziesii* QCAM7788, *Trametes menziesii* QCAM7790, and *Trametes meyenii* QCAM7785, which were selected for a 60-day soil microcosm experiment under controlled laboratory conditions. These strains removed over 96% of total petroleum hydrocarbons from contaminated soil, demonstrating high biodegradation efficiency. These results highlight the promise of native fungal strains as bioremediation agents for petroleum-contaminated soils. Further studies should focus on evaluating their performance under field conditions and their potential integration into large-scale remediation strategies.

## 1. Introduction

The oil industry accounts for approximately 30–34% of Ecuador’s total revenue and 11.3% of its GDP [1]. The activities associated with this industry, such as exploration, oil refining, oil spills, underground storage tank leaks, and industrial runoff/discharges, lead to the presence of petroleum hydrocarbons in the environment. These hydrocarbons include alkanes, alkenes, aromatics, and polyaromatics. Altogether, these compounds can be measured as Total Petroleum Hydrocarbons (TPH). Most of these substances are hydrophobic petroleum-derived compounds characterized by low density, limited emulsification capacity, and high resistance to biodegradation. They are highly polluting because of their persistence in the environment, especially in water and soil, affecting the flora and fauna, as well as human beings. Hydrocarbons have harmful properties and are considered carcinogenic, toxic, mutagenic, and teratogenic compounds [2].

Kuppusamy et al. (2019) [3] estimated that the Ecuadorian Amazon was contaminated with 714 million barrels worth of oil-polluted material from 1972 to 1993, which makes it the country with the largest oil spill pollution in the world. Decades of oil exploitation in the Ecuadorian Amazon have inflicted direct health consequences upon the population. Environmental contamination has triggered a health crisis, with communities experiencing a surge in illnesses ranging from memory loss problems and skin disorders to gastrointestinal issues. The toxic substances leaching into soil and water have exposed residents to carcinogens and heavy metals, resulting in elevated rates of cancers, birth defects, and other debilitating diseases [4]. The severe health impacts on Amazonian communities underscore the urgent need for immediate intervention and the implementation of sustainable, responsible resource management in oil exploitation.

Ecuador ranks sixth among the megadiverse countries worldwide [5]. These countries stand out for hosting a wide range of species of plants, animals, and other organisms, as well as the diversity of habitats in a particular territory. The conservation of these megadiverse countries is crucial to preserving the planet’s biological wealth. Ecuador has several national parks and protected areas that contribute to conservation efforts. Despite ongoing deforestation, the country harbors an extensive diversity of fungal species [6]. Until 2021, 6200 species of fungi were described out of approximately 96,000 fungal species estimated to exist in Ecuador [7]. In recent decades, the study of fungi has evolved, as well as their application in different areas such as medicine, plant disease biocontrol, biofertilizers, food industry, among other applications [8]. An important application field of the fungi kingdom is mycoremediation, which involves the use of fungal strains capable of degrading pollutants through their enzymatic activity and the production of secondary metabolites. These metabolites can enhance mycoremediation efficiency by acting as biosurfactants, increasing pollutant bioavailability, or by stimulating the growth and activity of coexisting pollutant-degrading microorganisms [9].

Fungi play a significant role in various ecosystems, serving as both decomposers and symbionts, due to their resilient morphology and versatile metabolic capabilities [10]. These attributes make fungi particularly well suited for mycoremediation purposes because they have the capability to remediate a wide range of pollutants such as pesticides, dyes, polycyclic aromatic hydrocarbons (PAH), polychlorinated biphenyls (PCBs), and hydrocarbons. They employ various mechanisms, including cellular absorption, chemical transformation of contaminants, and pollutant capture and retention [8]. Within the fungal kingdom, white rot fungi (WRF) play a central role in the biodegradation of lignin-rich material in natural environments, contributing significantly to global carbon recycling. They are considered the most efficient lignin degraders in nature [11].

Lignin is a biopolymer composed of aromatic units in the form of phenylpropanoids. The ability of WRF to degrade xenobiotic compounds is related to its capacity to degrade lignin [11]. WRF produces extracellular enzymes that catalyze the oxidative depolymerization of lignin. The main identified enzymes are manganese peroxidase (MnP), lignin peroxidase (LiP), and the oxidase laccase (Lac). These enzymes allow the oxidation of lignin through radical non-specific reactions. The induced formation of free radicals in biopolymers causes the destabilization of some covalent bonds, leading to the progressive breakdown of the macromolecule [12].

The treatment of contaminated soil by mycoremediation presents an environmentally and economically sustainable alternative compared to currently available methods [10]. Most pollutant-degrading organisms belong to the phylum Ascomycota and Basidiomycota [11]. In addition to the production of extracellular enzymes, these fungi share other important characteristics: low substrate specificity [13], high tolerance to pollutants due to their ability to perform extracellular degradation without cellular uptake, instead relying on external mechanisms such as biosorption on the surface of their cell wall or the production of extracellular enzymes to interact with and degrade pollutants; the structure of mycelium mirrors an efficient approach to foraging, combining dispersed exploratory growth under nutrient-poor conditions with extensive exploitative expansion in more favorable environments [11].

WRF has been widely studied for its ability to degrade a broad range of organic pollutants, including petroleum hydrocarbons. Among the most frequently investigated species are *Phanerochaete chrysosporium*, *Trametes versicolor*, *Pleurotus ostreatus*, *Ganoderma lucidum*, and *Irpex lacteus*, all of which have demonstrated high potential in the bioremediation of contaminated soils due to their robust metabolic activity and ecological adaptability [14]. These fungi have been successfully applied in both laboratory and pilot-scale studies, showing promising results in the degradation of hydrocarbons and polycyclic aromatic hydrocarbons (PAHs). However, while the literature highlights the potential of such basidiomycetes, there is a recognized lack of field trials and studies involving native Amazonian strains, particularly from Ecuador [15].

The objective of this work was to identify native Ascomycota and Basidiomycota strains from Yasuní National Park, in the Ecuadorian Amazon, with high (per)oxidase activity and potential for application in mycoremediation.

## 2. Materials and Methods

### 2.1. Chemicals

All the chemicals were of analytical grade or higher purity (Sigma-Aldrich (St. Louis, MO, USA) and Merck (Burlington, MA, USA)): malt extract agar (MEA 2%), gallic acid, Remazol brilliant blue R (RBBR), guaiacol, 2,2-azinobis-3-ethylbenzothiazoline-6-sulfonic acid (ABTS), acetone, hexane, sulfuric acid, hydrochloric acid, cyclohexane, potassium hydroxide, Tween 80 and all the inorganic salts. Filtered (0.45 µm Millipore-Merck, Burlington, MA, USA) diesel blend from an Ecuadorian gas station was used.

### 2.2. Fungal Strains Cultures and Identification

All the fungi used in this project (Table 1) were collected in the Yasuní National Park in August 2022. The isolation, purification, and molecular identification were carried out in the laboratory of mycology Nayón of the Pontificia Universidad Católica del Ecuador (PUCE). The complete isolation and purification procedure can be found in the Supplementary Materials (Table S1). Mycelia or blended mycelial suspensions were prepared according to Haroune et al. (2014) [16] and obtained on 2% MEA from fresh cultures of each fungal species assessed (see Supplementary Materials for details). The fungal strains were deposited in the Fungario QCAM PUCE. The strain *Trametes versicolor* (QCAM 778) was used as reference strain in all the experiments.

For DNA extraction and molecular identification, fresh mycelium of each strain was obtained directly from the culture for 14 days on 2% MEA. The Kit innuPREP Plant DNA (Analytikjena ™, Jena, Germany) was used according to the manufacturer’s instructions. The extracted DNA was amplified by PCR using the primers ITS5—5′GGAAGTAAAAGTCGTAACAAGG 3′ and ITS—4′ TCCTCCGCTTATTGATATGC 3′ [17]. The reactions were carried out according to Jarramillo-Riofrío et al. (2022) [18], with the following conditions: initial denaturation (98 °C, 5 min); followed by 40 cycles with denaturation (98 °C, 10 s), hybridization (55 °C, 10 s), extension (72 °C, 30 s) and final extension (72 °C, 5 min). The PCR product was purified by mixing 10 μL of each amplicon with four μL of Illustra Exoprostar 1-Step exonuclease (VWR International Eurolab S.L, Barcelona, Spain). The purification reaction was carried out for 30 min in the thermocycler. The purified PCR products were then sent for sequencing to Macrogen INC (Seoul, Republic of Korea). After molecular identification, three strains were identified as *Neopestalotiopsis clavispora*, *Clonostachys* sp., and *Nigrospora sphaerica*, and were not investigated further because they are plant pathogens and do not belong to the white rot fungi. All the following experiments were carried out using the 16 remaining strains (Table 1).

### 2.3. Influence of Temperature on Mycelial Growth

A plug of each culture grown on 2% MEA was inoculated in the center of a 90 mm Petri dish with 2% MEA. The inoculated Petri dishes were incubated at 24 °C and 30 °C for 8 days. The growth rate was measured by the increase in diameter of the colony. Three replicates of each culture were analyzed.

### 2.4. Influence of Diesel on Fungal Growth

Diesel and Tween 80 were filter-sterilized (Acrodisc filter 0.2 µm). Tween 80 was added first, followed by diesel at a final concentration of 0.1%*v*/*v* each in liquid (40 °C) MEA 2%. The suspension was prepared in a closed flask and maintained under magnetic stirring at 40 °C until homogeneous dispersion in MEA, before the suspension was poured into Petri dishes. A plug of each fungus grown on 2% MEA was inoculated at the center of the diesel/Tween/MEA Petri dishes. The 16 strains were also inoculated in MEA plates in the absence of diesel (control cultures). The cultures were incubated for 7 days at the best growth temperature (24 or 30 °C). The diameter of the fungal colony was measured after seven days. The mycelium growth inhibition (MGI) percentage was calculated as Equation (1) [19]. Three replicates of each culture were analyzed.

(1) MGI%=100 ∗ ((GC−GT)/(GC))

where

GC (growth control) represents the mean diameter of the fungi grown on MEA.
GT (growth treatment) represents the mean diameter of the three replicates of the fungi exposed to diesel.

### 2.5. Screening of Fungal Strains for Oxidative Enzymatic Activities

Qualitative enzymatic tests were performed by inoculating a 5 mm plug of mycelium (previously cultured for 14 days on 2% MEA) in the center of a 90 mm Petri dish containing the specific media described below. Three replicates of each culture were analyzed.

#### 2.5.1. RBBR Dye Discoloration Test

Laccase can decolorize RBBR [20]. The specific culture medium was made with 50 mg of RBBR per liter of MEA medium to detect a potential production of laccase (Lac). All fungal strains were incubated for 35 days at their best growth rate temperature on the RBBR MEA in Petri dishes. They were visually checked daily to determine the diameter of the fully discolored area in the culture medium.

#### 2.5.2. Gallic Acid Oxidation Test

In the presence of Lac, LiP, or MnP enzymes, gallic acid is oxidized to quinone-like products, including gallic acid-derived quinones and oligomers, resulting in a characteristic brown color [21]. The specific culture medium was made with 0.4 g of gallic acid per liter of MEA medium. Cultures in Petri dishes were incubated for 20 days at their best growth temperature (24 or 30 °C) and were visually examined daily to determine the formation of the brown color.

#### 2.5.3. Guaiacol Oxidation Test

Lac and peroxidases (Per) can oxidize guaiacol to the tetraguaiacol dimer that is detected as a reddish-brown color [22]. The specific culture medium was made with 0.2% (*v*/*v*) of guaiacol per liter of MEA medium. Cultures in Petri dishes were incubated for 20 days at the best growth temperature (24 or 30 °C) and were visually examined daily to determine the formation of the reddish-brown color.

### 2.6. Laccase Production in Liquid Culture

The enzymatic screening was performed in 100 mL Erlenmeyer flasks containing 50 mL of the basal medium of Tien & Kirk (1988) [23]. This basal medium stimulates ligninolytic enzymes. Its composition per liter is: 0.2 g ammonium tartrate, 3.28 g sodium acetate, 0.002 g thiamine, 2 g KH_2_PO_4_, 0.53 g MgSO_4_·7H_2_O, 0.1 g CaCl_2_, 0.001 g CuSO_4_, 0.005 g MnSO_4_, 0.0001 g H_3_BO_3_, 0.0001 g NaMoO_4_·2H_2_O, 0.001 g ZnSO_4_.7H_2_O, 0.001 g CoCl_2_, 0.01 g NaCl and 0.001 g FeSO_4_·7H_2_O. In addition, 4 g of wood chips (*Pinus patula*) were added as a carbon source. Sterile-filtered thiamine solution was added after the steam sterilization. The flasks were inoculated with 1 mL of the mycelium suspension of each strain (see Supplementary Materials for details). All experiments were performed in triplicate under controlled conditions (25 °C/150 rpm). The activity of laccase was monitored every two days for the first 20 days, with a final measurement taken on day 30.

### 2.7. Mycoremediation Assay Methodology

Five fungal strains were selected to perform a mycoremediation assay: *Ganoderma* cf. *parvulum* QCAM7791, *Trametes menziesii* QCAM7783, *Trametes menziesii* QCAM7788, *Trametes menziesii* QCAM7790, and *Trametes meyenii* QCAM7785.

#### 2.7.1. Contaminated Soil

A non-sterile, oil-contaminated soil collected close to an oil well in the Ecuadorian Amazon was used to study the potential of these fungi to remove TPH from soils of similar physicochemical characteristics. The soil had been previously treated by the oil company through a washing process using chemical products, including limonene, butyl glycol, sodium lauryl ether sulfate, and nonylphenol ethoxylate 9. After this treatment, the soil was placed in a biopile, where it remained for one year until it was sampled. The soil sample used in the present study was maintained under controlled humidity (30–50%) and temperature (20–25 °C) conditions until analysis. The initial characteristics of the soil before the remediation assay were measured: pH 7.4, conductivity 375 μS/cm, 3.86% C, 0.07 % N, total heterotrophic aerobic bacterial count: 27 × 10^3^ CFU/g-fresh soil, fungi count: 84 × 10^3^ CFU/g_wet_soil), TPH (C8-C40) concentration was 1.053 g/kg dried soil (reported per kg of initial dry soil), no Polycyclic Aromatic Hydrocarbon (PAHs) was detected. According to the values of apparent density and effective porosity, the soil was classified as clayey to sandy loam [24]. A detailed description of the methods employed is provided in the Supplementary Material.

#### 2.7.2. Organic Substrate for Fungal Growth

Industrial feed mix for rabbits (Cuy/conejos Engorde LB Bioalimentar Ecuador) was used as substrate for fungal growth in the remediation assay [25]. Studies suggest that optimal C/N for fungal growth ranges from 10 to 46 g_C/N [26,27,28]. The industrial feed mix for rabbits presents the best C/N ratio (29 g_C/N) when compared with C/N ratios of other substrates such as sawdust or maize stalks.

#### 2.7.3. Inoculum Preparation

The mycoremediation experiments were carried out with inoculation by mycelial pellets produced in liquid culture, following the method described by Haroune et al. (2014) [16]. The complete procedure can be found in the Supplementary Materials.

#### 2.7.4. Mycelium Development on Solid Organic Substrate

Cultures were performed in 100 mL glass bottles with blue GL45 caps (Boeco, Hamburg, Germany) containing 30 g of autoclaved (120 °C/45 min) industrial feed mix for rabbits moistened with 30 mL of sterile water. The substrate was homogenized by placing the bottles on a horizontal orbital shaker. Inoculation was carried out with 10 mL of homogenized mycelium suspension. The cultures were incubated under static conditions at ambient laboratory temperature and humidity, under controlled conditions for seven days.

#### 2.7.5. Mycelium Development on Polluted Soil

Ninety g of fresh contaminated soil were weighed in a 500 mL transparent PP, circular plastic container 125 × 55 mm (diameter × height, Plásticos Ecuador, Quito, Ecuador), then 30 g of mycelium colonized organic substrate was added and homogenized with the soil. For TPH removal efficiency determination, all final TPH concentrations were calculated and normalized to the initial dry mass of contaminated soil (90 g) to ensure direct comparability with initial TPH levels and controls without added substrate. For carbon balance analysis, all measured components were standardized to the same unit. These components included TPH, organic matter, fungal biomass, and mineralized carbon. All values were expressed per kilogram of total dry matter. For fungal treatments and negative controls, values were reported per kilogram of total dry matter (soil + substrate combined), and the total dry matter consisted of both soil and substrate combined. For controls without added substrate (soil d0 and soil d60), values were expressed per kilogram of dry soil only to reflect the overall carbon dynamics of the system. Containers were covered with a vented lid (25 cm^2^ covered with sterile gauze permeable to gas) and incubated at 28 °C under static conditions in the dark. The cultures were performed in triplicate. Two types of non-inoculated soil blanks were used as negative controls: the first one with non-colonized organic substrate (Negative control) and the second one without organic substrate and no inoculum (Soil). In parallel, positive control using *Trametes versicolor* was included. After two months of incubation at 28 °C, the cultures were sacrificed, homogenized, and sampled for analysis. All treatments, including the controls, were performed in triplicate.

### 2.8. Analysis Methods

#### 2.8.1. Laccase Activity Measurement

The activity of laccase in liquid culture was monitored every two days for 20 days. 10–100 µL of the centrifuged/filtered culture supernatant was added to 490–400 µL of 0.1/0.2 M citrate/phosphate buffer at pH 4. 500 µL of a 0.5 mM ABTS solution was added, and the absorbance at 420 nm was monitored for 2 min with a UV-Vis spectrophotometer Thermo Orion Aqua Mate 7000, Thermo, Waltham, MA, USA. The evolution of the absorbance was converted to activity using a molar extinction coefficient of the radical cation ABTS ε: 36,000 Abs/(M^−1^·cm^−1^). An additional activity measurement was performed on day 30 with a fresh ABTS solution.

#### 2.8.2. Characterization of the Untreated and Treated Soil

The initial soil sample (i.e., collected after the industrial biopile treatment) was characterized for the following parameters: effective porosity, apparent density [29] (USDA, 2014), pH [30] (EPA method 9045D), humidity [31] (ASTM D2216-98 1998), organic matter [32] (ASTM F1647-11 2018), nitrogen [33,34] (EN 13342/DIN ISO 11261), total petroleum hydrocarbons TPHGC [35] (EPA method 8015D, by GC-FID), polycyclic aromatic hydrocarbons (PAHs) [36] (AOAC Method 2007.01.2005), elemental analysis [37] (EPA method 6020B), and soil respiration [38] (USDA 1999). A detailed summary of each procedure can be reviewed in the Supplementary Materials. After the two months of mycoremediation assay, the same parameters were measured in the treated soils except for effective porosity and apparent density. For the assessment of TPH removal percentages, final TPH concentrations in treated samples were recalculated and are consistently reported as g/kg of the initial dry soil mass to accurately represent the degradation efficiency. Other parameters, such as organic matter, nitrogen, and microbial populations, are reported per kg of total dry mixture (soil + substrate) for treatments where substrate was added, and per kg of dry soil for controls without added substrate (soil d60).

#### 2.8.3. Ergosterol Quantification

Ergosterol was extracted from the treated soil and analyzed as previously described by Borràs et al. (2010) [39]. In addition, a fortified soil sample was prepared with a final concentration of 0.1 mg/g to validate the extraction and quantification method. Briefly, 0.5–0.8 g of the soil (with its humidity) was extracted for 90 min at 70 °C (sonicating for the first 15 min) with a mixture of 1 mL cyclohexane and 3 mL of a KOH/methanol solution (10% *w*/*v*). Then one mL of distilled water and 2 mL of cyclohexane were added. The tube was vortexed for 30 s and centrifuged at 3500 rpm for 5 min. The organic phase was recovered, and the aqueous phase was washed twice with 2 mL cyclohexane. The organic phases were pooled and evaporated to dryness using a Speed/Vac SPD140DDA. (Thermo Fisher Scientific, Waltham, MA, USA). The residue was dissolved in 0.5 mL methanol for 15 min at 40 °C, vortexed for 30 s, and centrifuged at 6000 rpm for 3 min. Finally, the resulting supernatant was filtered (PVDF membrane 0.22 µm) and transferred to amber glass vials. They were analyzed in a Waters 1525 HPLC (High-Performance Liquid Chromatography) (Waters Corporation, Milford, MA, USA), equipped with an UV detector set at 282 nm, using a reverse phase column Xbridge C18 (4.6 × 150 mm, 5 μm). Methanol was used as mobile phase at a constant flow of 1.4 mL·min^−1^.

### 2.9. Assessment of Microbial Populations

The soil microbial population was assessed at the start and at the end of the 2-month mycoremediation assay. All the media used for this assessment were from Sigma Aldrich. For the assessment, 10 g of soil samples (with their humidity) were collected and serially diluted with 0.1% peptone (BD DIFCO) in water in a tenfold series and spread over selective media [40]. Table S4 shows information about the dilutions used. The total bacterial population was assessed on a nutrient agar medium with nystatin (50 mg/L) to prevent the growth of fungi. Actinomycetes were assessed on ISP2 medium, as described by Karkouri (2019) [41]. Bacteria capable of growth on King B agar medium and fluorescing under UV light were isolated as described by Kotasthane (2019) [42]. Phosphate-solubilizing bacteria were isolated on Pikovskaya medium [43]. The yeast and mold populations were isolated using Rose Bengal agar medium supplemented with nalidixic acid (50 mg/L), as detailed by Chumchalova and Kubal (2020) [44].

### 2.10. Carbon Mass Balance Calculation

The carbon balance of the treatments was determined using the measured values of soil organic carbon, hydrocarbons, and ergosterol. The values of hydrocarbons at initial and final time were converted to carbon using the reference molecular weight of diesel (C_12_H_26_). The carbon content present in the fungal biomass was calculated by converting the measured concentration of ergosterol, using the coefficient reported by Djajakirana et al. (1996) [45] (Equation (2)). Concentrations of organic carbon, (OC), attributed to organic matter in soil and organic substrate, were measured initially and at the end of the 60-day incubation period. Then, the carbon mineralized during the bioremediation process was calculated by the difference between initial and final organic carbon at the end of the 60-day incubation period (Equation (3)).

(2) $$Carbon\;content\;in\;fungal\;biomass\;\left(mg\right)=\left(ergosterol\;mg\right)\times \frac{0.46}{0.005}\;$$

(3) Mineralized Carbon gkg soil=OCinitial−OCafter treatment

### 2.11. Statistical Analysis

The statistical analysis was performed using R version 4.4.1 (14 June 2024) with R-studio interface. Normality was assessed with the Shapiro–Wilk test and homogeneity of variances with Levene’s test. Since the assumptions for parametric tests were not met, non-parametric methods were applied. The Kruskal–Wallis test was used to detect significant differences among treatments. For all comparisons, post hoc comparisons using Dunn with Benjamini–Hochberg correction to control the false discovery rate (FDR). Statistical significance was set at α = 0.05.

## 3. Results

### 3.1. Fungal Strains Identification

DNA-based identification revealed that 16 out of the 19 tested strains belong to the phyla Ascomycota or Basidiomycota (Table 1). The other three strains, i.e., *Neopestalotiopsis clavispora*, *Clonostachys* sp., and *Nigrospora sphaerica*, were excluded from further analysis because they are plant pathogens and do not exhibit ligninolytic traits characteristic of WRF. The sequences obtained in this study were submitted to NCBI (Table 1).

### 3.2. Diesel and Temperature Influence on Fungal Growth

**Figure 1:**
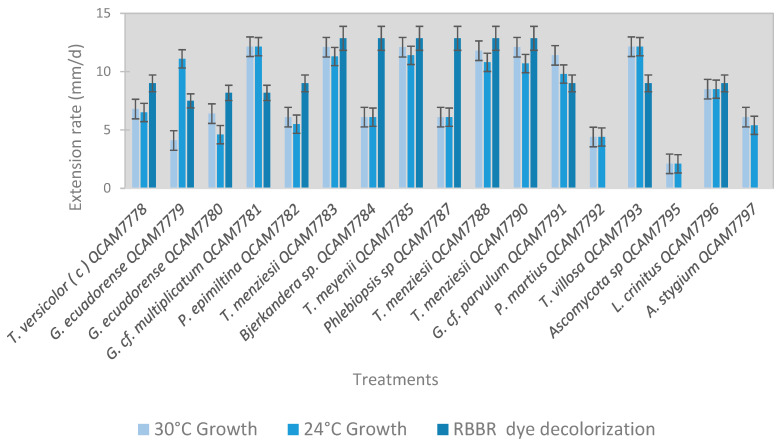
Fungal growth rates at 24 °C and 30 °C, and RBBR dye discoloration test (attributed to laccase activity). Rates expressed in mm/d correspond to the extension of the diameter of the area of the agar plate colonized by the mycelium (growth) and by dye discoloration (laccase activity). Error bars represent standard deviation (n = 3).

All strains, except *Ganoderma ecuadorense* QCAM 7779, exhibited similar growth rates at both 24 °C and 30 °C temperatures (Figure 1). Table 1 summarizes the diesel tolerance of each strain. Based on the percentage of mycelial growth inhibition (%MGI), nine strains were classified as not significantly affected by diesel exposure, showing less than 15% MGI. Two strains exhibited strong growth inhibition, with MGI values exceeding 50%. The remaining five strains showed moderate inhibition, with MGI values ranging from 18% to 36%, like the reference strain *T. versicolor*.

### 3.3. Screening of Fungal Strains for Oxidizing Enzymatic Activities

Six strains presented a discolored agar area extension rate of 13 mm/d (decolorizing the entire Petri dish within 7 days), which was superior to the 9 mm/d of the *T. versicolor* (the reference strain); the seven remaining strains presented rates between 8 and 9 mm/d (decolorizing the entire Petri dish within 12 days). Two of the three strains that did not degrade the RBBR belong to the phylum Ascomycota. The third strain, despite being a basidiomycete, did not show enzymatic activity towards laccase also in the liquid culture assay.

Table 1 shows that 10 out of the 16 strains were positive for the guaiacol oxidation assay, suggesting the presence of laccase and/or peroxidase activity [46]. *Ganoderma* cf. *parvulum* was the only strain that did not show a positive result for the phenol oxidases test. *Trametes versicolor* showed positive results in both tests. A variability is observed between the activity patterns obtained. *Ganoderma ecuadoriense* presented negative results for Laccase and Peroxidase guaiacol test, while presenting positive results for the Lac dye discoloration test. Similar results were observed for *Ganoderma* cf. *multiplicatum*, *Bjerkandera* sp., *Trametes villosa*, *Ascomycota* sp., and *Lentinus crinitus*. On the other hand, the strains *Hornodermoporus martius* and *Annulohypoxylon stygium* presented positive results for the guaiacol test and negative for laccase dye discoloration.

### 3.4. Laccase Production Under Submerged Culture Conditions

**Figure 2:**
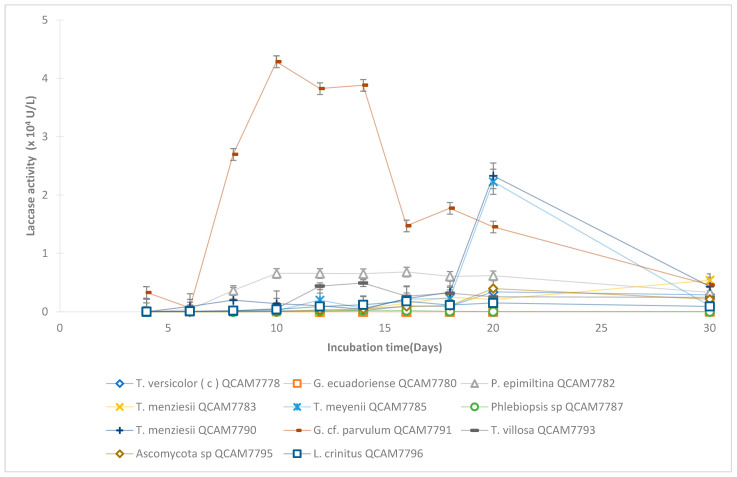
Evolution of soluble Laccase activity (ABTS assay) over 30 days in submerged cultures. Error bars: standard deviation (n = 3).

Laccase activity varied significantly among the 16 strains tested (Figure 2). *Ganoderma* cf. *parvulum* showed the highest laccase activity (4.3 ± 0.1) × 10^4^ U/L on day 10, while *Ganoderma ecuadoriense* showed no activity during the 20-day assay but exhibited low activity on day 30 (40 ± 5 U/L). Differences in time to achieve the highest activity were observed. The strains that achieved the highest activity earlier were: *Ganoderma* cf. *parvulum* and *Trametes villosa* at day 10 and 12, respectively. *Lentinus critinus*, *Porogramme epimiltina*, and *Phlebipsis* sp. achieved the highest activity on day 16, *Trametes versicolor* (the reference strain, (3 ± 0.5) × 10^3^ U/L), *Ascomycota* sp., *Trametes menziesii*, and *Trametes meyenii* at day 20.

### 3.5. Mycoremediation Assay Results

Five fungal strains were selected based on enzymatic activity, low % MGI, and high laccase dye discoloration rate.

**Figure 3:**
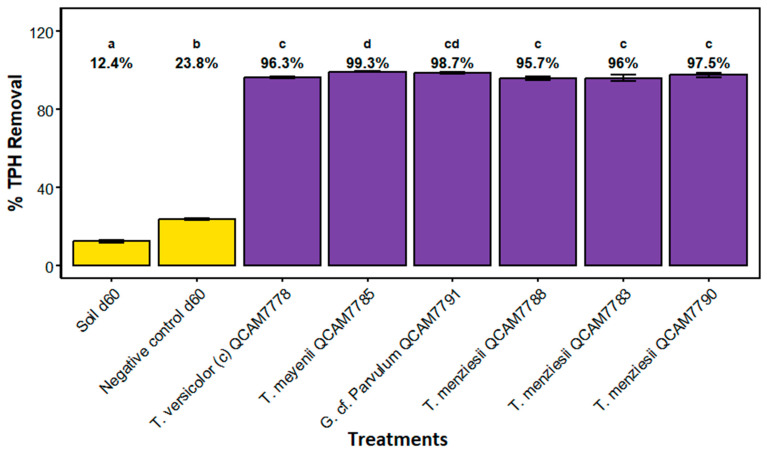
Percentage of TPH removal after 60 days of incubation with the specified strain. Calculated based on the initial TPH content in the dry soil. Soil d60: soil incubated without substrate and inoculum, negative control d60: soil with substrate but without inoculum. Letters above treatments indicate statistical groups; treatments sharing the same letter are not significantly different (Kruskal–Wallis test followed by Dunn’s post hoc test with Benjamini–Hochberg correction, *p* < 0.05, n = 3).

The analysis of TPH removal efficiency revealed significant differences between treatments (Figure 3). Four distinct statistical groups were identified based on TPH removal performance. The highest removal efficiency was achieved by *T. meyenii* QCAM7785 (group d) with 99.3 ± 0.3% removal, followed by *G.* cf. *Parvulum* QCAM7791 (group cd) with 98.6 ± 0.9% removal. A large intermediate group (group c) included *T. versicolor* QCAM7778 (96.3 ± 1%). *T menziesii* QCAM7783 (96.0 ± 2.9%). *T. menziesii* QCAM7790 (97.5 ± 2.2%), and *T. menziesii* QCAM7788 (95.7% ± 1.5%), all achieving removal efficiencies above 95%. In contrast, both control treatments showed significantly lower performance: the negative control d60 (group b) achieved 24.0 ± 0.8% removal, while the soil control d60 (group a) showed the lowest removal efficiency at 12.4 ± 1.4%. The difference between fungal treatments and controls exceeded 70 percentage points, demonstrating the substantial bioremediation potential of the fungal isolates tested. Representative chromatograms of negative control and the treatment with *G.* cf. *parvulum* are shown in Figure S1.

**Figure 4:**
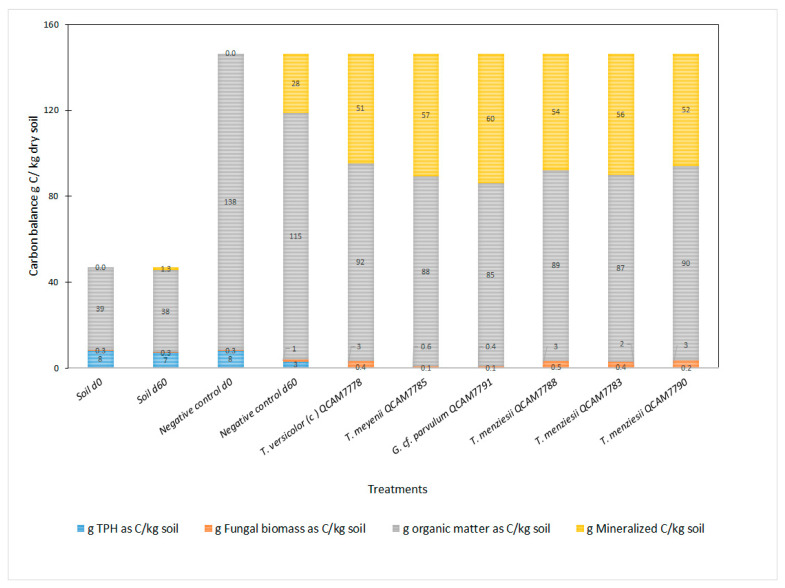
Carbon balance of mycoremediation treatments. Values are expressed per kilogram of total dry matter (soil + substrate) for fungal treatments and negative control d60, and per kilogram of dry soil for Soil d0 and Soil d60.

Figure 4 shows the estimated carbon balance. The results from the five different fungal treatments are similar. All treatments were effective in hydrocarbon degradation, with residual TPH carbon values ranging from approximately 0.04 to 0.01 g C/kg of total dry matter, similar levels of mineralized carbon (51–60 g C/kg dry matter), and remaining organic matter carbon (85 to 92 g C/kg). The fungal biomass was low for all treatments (<3 g C fungi/kg dry matter).

**Figure 5:**
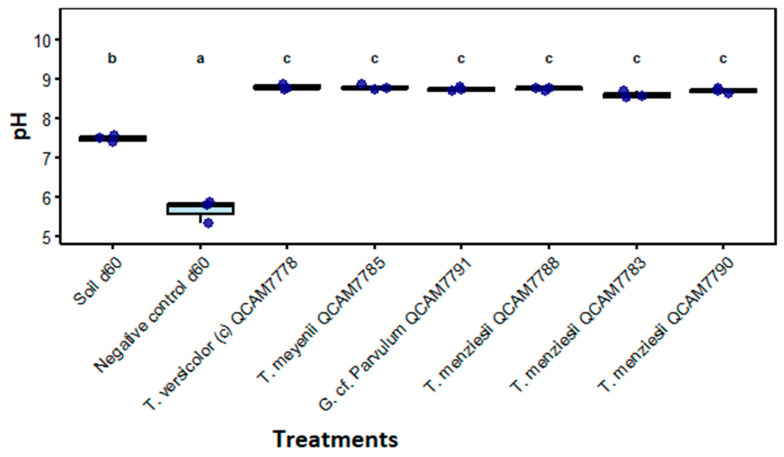
pH levels after 60 days of incubation with the specified strain. Letters above treatments indicate statistical significance groups; treatments with the same letter are not significantly different (Kruskal–Wallis, *p* < 0.05, n = 3).

pH values presented highly significant differences between treatments and controls (*p* < 0.001), showing three clearly differentiated groups. All filamentous fungi treatments (group c) alkalinized the medium to pH values between 8.59 and 8.78. The soil d60 (group b) maintained a pH close to neutrality (7.48 ± 0.09), while the negative control (group a) showed acidic conditions (5.66 ± 0.28) (Figure 5).

**Figure 6:**
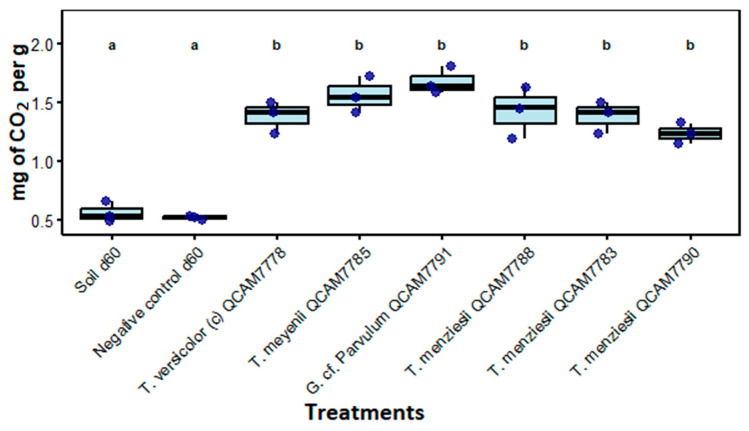
Soil respiration measured as mg of CO_2_ per g after 60 days of incubation with the specified strain. Letters above treatments indicate statistical significance groups; treatments with the same letter are not significantly different (Kruskal–Wallis, *p* < 0.05, n = 3).

Soil respiration measured as mg of CO_2_ per g showed significant differences between treatments (*p* < 0.001). All fungal treatments (group b) demonstrated significantly higher respiratory activity compared to controls, with mean values ranging from 1.2 to 1.7 mg CO_2_/g of wet soil; no significant differences were observed among fungal species. Controls (group a) showed significantly lower CO_2_ values: Soil d60 (0.56 ± 0.09 mg CO_2_/g) and Negative control d60 (0.52 ± 0.02 mg CO_2_/g) (Figure 6).

**Figure 7:**
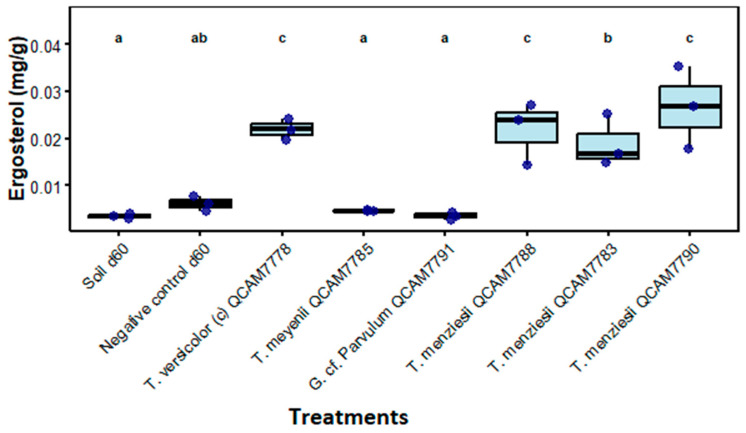
Ergosterol values mg/g after 60 days of incubation with the specified strain. Letters above treatments indicate statistical significance groups; treatments with the same letter are not significantly different (Kruskal–Wallis, *p* < 0.05, n = 3).

Statistical analysis reveals significant differences in ergosterol contents between treatments (*p* < 0.001). Fungal treatments showed considerably higher values than controls. *T. menziesii* QCAM7790 (0.027 ± 0.009 mg/g), *T. versicolor* QCAM7778 (0.022 ± 0.002 mg/g), and *T. menziesii* QCAM7788 (0.022 ± 0.007 mg/g) presented the highest ergosterol concentrations (group c). *T menziesii* QCAM7783 showed intermediate values (group b, 0.019 ± 0.005 mg/g), while controls and other fungal species presented lower concentrations (group a): Soil d60 (0.003 ± 0.0005 mg/g), Negative control d60 (0.006 ± 0.002 mg/g), *T. meyenii* QCAM7785 (0.005 ± 0.0002 mg/g), and *G.* cf. *Parvulum* QCAM7791 (0.003 ± 0.0008 mg/g) (Figure 7). Ergosterol control samples achieved recovery percentage between 65–71%.

### 3.6. Assessment of Microbial Population

**Figure 8:**
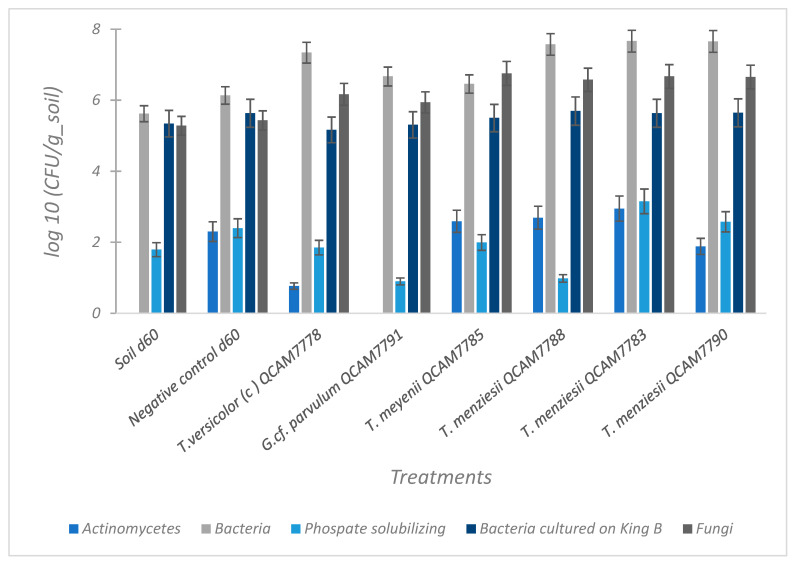
Microbial populations (log CFU/g wet soil) detected in soil samples after 60 days of incubation with different treatments. Different microbial groups were assessed on selective media: total bacteria on nutrient agar with nystatin, actinomycetes on ISP2 medium, bacteria grown on King B agar, phosphate-solubilizing bacteria on Pikovskaya medium, and yeasts/molds on Rose Bengal agar with nalidixic acid. Error bars represent standard deviation (n = 3).

Figure 8 compares the microbial populations detected after each treatment, the soil d60, and the negative control d60. Overall, the five fungal treatments showed a consistent dominance of bacterial populations, while fungal populations showed high variability; actinomycetes remained relatively stable, bacteria cultured on King B varied among treatments, and phosphate-solubilizing bacteria displayed the lowest levels. These data are consistent with the results from the soil respiration test, which indicated an increase in respiration for all treatments compared to the values obtained for soil d60 and the negative control d60.

## 4. Discussion

### 4.1. Diesel and Temperature Influence on Fungal Growth and Enzymatic Activity

The differences in growth rates under optimal temperature conditions are likely influenced by the genetic makeup of the fungi, which is shaped by the climatic and geographical features of the location where the fungi were originally isolated [47]. The results obtained are consistent with the temperature regime of Yasuní National Park, where day and night temperatures typically range between 24 and 27 °C year-round. Several studies also reported the optimal growth range for Ascomycota and Basidiomycota fungi between 25 and 30 °C [9,48,49].

The results of the oxidizing enzymatic activities showed high variability, which may be a consequence of the inherent complexity of fungal enzyme systems and semiquantitative nature of plate-based assays [49,50]. These screening methods, while useful for initial assessment, have recognized limitations in distinguishing between different oxidative enzymes and may not reflect optimal conditions for each species [51,52]. For instance, some species predominantly rely on laccases, while others utilize peroxidases or other oxidases to break down lignocellulosic or phenolic materials, highlighting the diverse enzymatic strategies employed by different fungal species to perform similar metabolic functions [49].

The discrepancy observed between RBBR decolorization and specific enzyme tests (laccase, peroxidase) in some strains suggests the involvement of additional oxidative enzymes not detected by our screening protocols. This phenomenon has been documented in other studies, where alternative enzymes such as versatile peroxidases or other oxidoreductases contribute to dye decolorization [51,53]. The high variability of results in the screening of ligninolytic activities is consistent with other studies that also show a high variability in ligninolytic activity among distinct species of the same genus and even between strains of the same species [54]. The agar plate assays are useful tests for initial screening, but they need to be complemented with additional analyses.

The laccase activities for *T. versicolor* (3 ± 0.5 × 10^3^ U/L) coincide with values reported in other studies, 2500–3200 U/L under similar cultivation conditions [52,55]. The enzymatic activity of *Trametes versicolor* is one of the most studied because it presents high laccase activity; in this study, three strains (*G.* cf. *parvulum*, *T. menziesii*, and *T. villosa*) demonstrated laccase activities exceeding that of *T. versicolor* by 2–14 fold, with *G.* cf. *parvulum* achieving the highest activity (4.3 ± 0.1 × 10^4^ U/L). This enhanced enzymatic capacity suggests these native strains may possess evolved adaptations to local environmental conditions and substrates.

The enzymatic activity detected in this study can be linked to the degradation of petroleum hydrocarbons through oxidative pathways. As previously discussed, these ligninolytic enzymes can generate reactive radicals that initiate the breakdown of aliphatic compounds, ultimately leading to their transformation into less complex molecules and, in some cases, complete mineralization [56,57]. The degradation of aliphatic hydrocarbons in the C8–C40 range analyzed here may therefore be partly explained by the action of these enzymes. Although additional mechanisms such as biosurfactant production or cytochrome P450-mediated oxidation have been proposed in the literature [58,59,60], these were not evaluated in the present study.

### 4.2. Mycoremediation Assay

The five assessed strains achieved TPH removal efficiencies ranging from 96% to 99%, significantly outperforming both the negative control (24 ± 2%) and natural attenuation (12 ± 2%) (*p* < 0.001, Kruskal–Wallis test). These results align with literature that highlights the effectiveness of species of *Trametes* and *Ganoderma* in degrading petroleum hydrocarbons, attributed to their ligninolytic capabilities [11,61].

The TPH removal observed in soil d60 and Negative control d60 is attributed to natural attenuation. In the case of the negative control, the greater removal likely occurred because the soil was not sterilized, suggesting that native microorganisms benefited from the substrate, although they were not as effective as the fungi added in the other treatments. Additionally, the residual chemical surfactants from the oil company’s previous washing treatment, as described in Section 2.7.1, might have further enhanced the bioavailability and subsequent degradation of TPH in all treatments, contributing to the observed high removal efficiency.

The high efficiency of the fungal strains assessed in degrading TPH is intrinsically linked to their metabolic pathways for breaking down complex organic matter. WRF, including the *Ganoderma* and *Trametes* species studied here, primarily utilize their ligninolytic enzyme system (e.g., laccases and peroxidases) for the degradation of lignin and other recalcitrant organic compounds [11,12,62]. In the context of Mycoremediation, the organic substrate (rabbit feed) served as a readily available carbon nutrient source, promoting fungal growth and inducing the production of these extracellular enzymes [25,26,27,28]. These non-specific enzymes then facilitate the biotransformation of petroleum hydrocarbons, while the fungi metabolize the more accessible organic matter [9]. This interrelationship explains the simultaneous degradation of both organic matter and TPH in the treatments. All fungal treatments show similar levels of mineralized carbon (51–60 g C/kg dry soil), indicating high metabolic activity and efficient conversion of TPH and organic matter into CO_2_ and other end products. The negative control d60 exhibits intermediate values; this suggests some use of the initial organic matter provided as substrate by the native soil microorganisms.

The proportion of organic matter remained relatively stable, ranging from 85 to 92 g C/kg_soil across treatments. The initial organic matter content was 138 g C/kg (Negative control d0). By day 60, the organic matter content in the negative control was reduced to 115 g C/kg of soil. This reduction aligns with the natural microbial activity previously discussed. These results indicate that the treatments tested demonstrated greater mycoremediation efficiency, achieving a TPH removal rate four times higher, with approximately doubling the organic matter consumption compared to the negative control d60.

The fungal biomass, measured as ergosterol at the end of the two-month assay, represented the total accumulated growth during the treatment period and was low for all treatments (<3 g C/kg dry soil). Interestingly, there was a significant difference in fungal biomass production between the strains (Figure 7). These observed differences can be explained by several factors:

(1) Metabolic strategy differences: High-efficiency strains may prioritize extracellular enzyme production and substrate mineralization over biomass accumulation [61,63]. This strategy is characteristic of primary decomposer fungi that rapidly process available substrates. In this study, all fungal species demonstrated similar respiratory activity (1.2–1.7 mg CO_2_/g) and TPH removal rates, indicating that while the pathways could differ, their metabolic efficiency remains consistent.
(2) Ecological adaptation: as primary colonizers of lignocellulosic materials, these species evolved to rapidly process substrates with minimal carbon retention, maximizing decomposition rates rather than biomass yield [64].
(3) Microbial competition appears to play a key role in shaping fungal dynamics: In this study, bacterial populations maintained high levels across all treatments, suggesting a dominant presence that could suppress fungal growth [65].
(4) Finally, fungi may be adopting a strategy focused on functional specialization rather than growth, allocating resources to the production of specific extracellular enzymes involved in TPH degradation. Degradation rates tend to correlate more closely with extracellular protein concentration and enzymatic activity than with overall fungal biomass. This could be related to the differences in bacteria cultured on King B and phosphate-solubilizing populations observed in this study, suggesting that each inoculated fungal species creates a distinct microenvironment, differentially influencing the compositions and function of the associated microbial communities [66].

These findings are consistent with previous studies that have demonstrated the capacity of these fungi to degrade organic pollutants in soils, achieving significant mineralization without corresponding increases in fungal biomass [63,67].

pH values showed significant differences among the treatments (Figure 5 and Figure S2A). Fungal treatments exhibited higher pH values (7.8–8.2) compared to the controls (6.9–7.4). While the rabbit feed used as substrate contained substantial concentrations of calcium and magnesium (see Table S5), which are known to influence soil alkalinity through interactions with organic matter, the observed pH increase likely reflects multiple factors beyond cation release from mineralization processes [68,69,70].

## 5. Conclusions

This study successfully identified and characterized 16 native fungal strains from the phyla Ascomycota and Basidiomycota, isolated from Ecuador’s Yasuní National Park. All strains exhibited (per)oxidase activity in at least one qualitative assay. Five of these strains showed promising potential in the degradation of petroleum hydrocarbons under laboratory conditions, with removal efficiencies comparable to that of the model strain *Trametes versicolor*. While these findings are encouraging, they are based on preliminary laboratory tests. Further research is needed to validate these results under field conditions and to account for environmental variables such as soil composition and pollutant complexity.

## Figures and Tables

**Table 1:** Results of the RBBR decolorization test, laccase and peroxidase Guaiacol test, phenol oxidases test, and diesel tolerance test (%MGI) for each tested fungal strain.

Fungal PUCE Code	Genbank	Fungal Species	Phylum	RBBR Test	Lac and Per Guaiacol Test	Phenol Oxidases Test	Growth Inhibition by 0.1% Diesel (%MGI)
QCAM7778 MUCL11665	PQ660253	*Trametes veriscolor* (reference strain)	Basidiomycota	+	+	B	18
QCAM7779	PQ660252	*Ganoderma ecuadorense*	Basidiomycota	+	−	Y	22
QCAM7780	PQ328986	*Ganoderma ecuadorense*	Basidiomycota	+	+	YB	51
QCAM7781	PQ328985	*Ganoderma* cf. *multiplicatum*	Basidiomycota	+	−	Y	0
QCAM7782	PQ328987	*Porogramme epimiltina*	Basidiomycota	+	+	YB	0
QCAM7783	PQ328988	*Trametes menziesii*	Basidiomycota	+	+	Y	0
QCAM7784	PQ328989	*Bjerkandera* sp.	Basidiomycota	+	−	B	0
QCAM7785	PQ328990	*Trametes meyenii*	Basidiomycota	+	+	YB	36
QCAM7787	PQ328984	*Phlebiopsis* sp.	Basidiomycota	+	+	B	0
QCAM7788	PQ328983	*Trametes menziesii*	Basidiomycota	+	+	Y	0
QCAM7790	PQ328982	*Trametes menziesii*	Basidiomycota	+	+	YB	27
QCAM7791	PQ328981	*Ganoderma* cf. *parvulum*	Basidiomycota	+	+	-	0
QCAM7792	PQ328980	*Hornodermoporus martius*	Basidiomycota	−	+	B	20
QCAM7793	PQ328979	*Trametes villosa*	Basidiomycota	+	−	YB	29
QCAM7795	PQ328978	*Ascomycota* sp.	Ascomycota	−	−	YB	54
QCAM7796	PQ328977	*Lentinus crinitus*	Basidiomycota	+	−	YB	0
QCAM7797	PQ328976	*Annulohypoxylon stygium*	Ascomycota	−	+	Y	0

RBBR decolorization test: + Medium discoloration in 35 days or less; − no medium discoloration. Lac and Per guaiacol test: + reddish-brown color reaction; − no reaction. Phenol oxidases test: B, brown; YB, yellowish brown; and Y, yellow. %MGI (Mycelium growth inhibition with 0.1% diesel, as compared to no diesel).

## Data Availability

The original contributions presented in the study are included in the article/Supplementary Material; further inquiries can be directed to the corresponding author.

## Supplementary Materials

The following supporting information can be downloaded at https://www.mdpi.com/article/10.3390/jof11090651/s1. Figure S1: Chromatograms of one replicate of negative control and *Ganoderma* cf. *parvulum* after the 60-day incubation period. Figure S2: Relation between TPH removal yield with pH, % N, soil respiration (mg of CO_2_/g) and Fungal biomass (mg/g); Table S1: Sample locations and the original substrates of the collected specimens. Table S2: TPHs analysis GC conditions; Table S3: Multielemental soil digestion conditions; Table S4: Population assay mediums and dilutions; Table S5: Rabbit food multielemental analysis.
