# Supplementary material for: Mycoremediation of Petroleum-Contaminated Soil Using Native Ganoderma and Trametes Strains from the Ecuadorian Amazon
Source: J Fungi (Basel). 2025 Sep 2;11(9):651. doi: 10.3390/jof11090651 (PMC12470330; doi:10.3390/jof11090651)
Supplement: Supplementary file 1 [file jof-11-00651-s001.zip › jof-3755468-supplementary.pdf]

Journal of *Fungi*

Supplementary Materials

Materials and methods

Fungal Isolation

Fruiting bodies with typical basidiomycete features were collected, surface-rinsed three times with sterile water, and dried with sterile gauze. Hymenial tissue was aseptically excised and transferred to 2% MEA supplemented with chloramphenicol (0.1 g/L). Each sample was plated in triplicate and incubated at 25°C for 7 days, with daily monitoring. Emerging mycelia were subcultured from the colony margin onto fresh medium, repeating the process until pure cultures were obtained. Table S1 shows sampling locations and the original susbstrates of the collected specimens.

Table S1. Sample locations and the original substrates of the collected specimens

Fungal PUCE code	Genbank	Fungal species	**Sampling location**	**Substrate**
QCAM7779	PQ660252	*Ganoderma ecuadorense*	*0,66716° S, 76,40398° O*	*Decaying wood*
QCAM7780	PQ328986	*Ganoderma ecuadorense*	*0,66716° S, 76,40398° O*	*Decaying wood*
QCAM7781	PQ328985	*Ganoderma cf.multiplicatum*	*0,66716° S, 76,40398° O*	*Decaying wood*
QCAM7782	PQ328987	*Porogramme epimiltina*	*0,66716° S, 76,40398° O*	*Decaying wood*
QCAM7783	PQ328988	*Trametes menziesii*	*0,66716° S, 76,40398° O*	*Balsa (Ochroma pyramidale)*
QCAM7784	PQ328989	*Bjerkandera sp.*	*0,66716° S, 76,40398° O*	*Decaying wood*
QCAM7785	PQ328990	*Trametes meyenii*	*0,66716° S, 76,40398° O*	*Caoba (Swietenia macrophylla)*
QCAM7787	PQ328984	*Phlebiopsis sp*	*0,66716° S, 76,40398° O*	*decaying wood*
QCAM7788	PQ328983	*Trametes menziesii*	*0,66716° S, 76,40398° O*	*Caoba (Swietenia macrophylla)*
QCAM7790	PQ328982	*Trametes menziesii*	*0,66716° S, 76,40398° O*	*Decaying wood*
QCAM7791	PQ328981	*Ganoderma cf. parvulum*	*0,66716° S, 76,40398° O*	*Decaying wood*
QCAM7792	PQ328980	*Hornodermoporus martius*	*0,66716° S, 76,40398° O*	*Decaying wood*
QCAM7793	PQ328979	*Trametes villosa*	*0,66716° S, 76,40398° O*	*Decaying wood*
QCAM7795	PQ328978	*Ascomycota sp*	*0,66716° S, 76,40398° O*	*Forest leaf litter*
QCAM7796	PQ328977	*Lentinus crinitus*	*0,66716° S, 76,40398° O*	*Decaying wood*
QCAM7797	PQ328976	*Annulohypoxylon stygium*	*0,66716° S, 76,40398° O*	*Decaying wood*

Mycelium suspension preparation

Five 8 mm^2 plugs of each of the five strains, previously cultured for 14 days on 2% MEA Petri dishes, were inoculated into 500 mL Erlenmeyer flask containing 250 mL of 2 % malt extract broth. After seven days of cultivation at 26°C and 135 rpm, the suspension was filtered using a sterile Corning© cell strainer 100 µm pore size, and the fungal biomass was rinsed with a sterile 0.89% NaCl solution. The fungal biomass was then blended using an Omni mixer homogenizer, in the saline solution and stored at 4°C. This mixture is referred to as "clamps suspension". Two milliliters of this blended suspension were transferred to a 500 mL Erlenmeyer flask containing 250 mL of 2 % malt extract broth. After seven days of cultivation at 26°C and 135 rpm, the mixture was filtered using a sterile Corning© cell strainer 100 µm pore size, and the fungal biomass was rinsed with sterile 0.89% NaCl solution. The fungal biomass was blended in the saline solution and stored at 4°C. This last mixture is referred as "mycelium suspension", this suspension was used within seven days.

Soil pH and conductivity

To determine pH and conductivity, a portable multiparameter device (HACH HQ40D) was used, calibrated with pH standards of 4.01, 7.00, and 10.01, and a conductivity standard of NaCl 1000 µS/cm. A 20 g soil sample was mixed with 50 mL of water and mechanically stirred for 5 minutes. The mixture was then allowed to settle for one hour, after which the pH and conductivity were measured by inserting the respective electrode into the supernatant.

Soil humidity

The water content in the contaminated soil was quantified using the gravimetric method. A 10 g soil sample in a tared crucible was dried in an oven at 105 °C for 24 hours, then cooled to room temperature in a desiccator. The humidity percentage was determined based on the weight difference.

Soil organic matter

The presence of organic carbon was analyzed using a gravimetric method which involves the volatilization of all forms of organic carbon in the contaminated soil sample. First, a 10 g soil sample was dried at 105 °C in a tared crucible for 24 hours to remove moisture. Afterward, the sample was calcined in a muffle furnace at 550 °C for 4 hours. The percentage of organic matter was then determined as the weight loss. The percentage of organic carbon was calculated by dividing the organic matter value by 1.724 g_organic_matter / g_carbon [50].

Soil nitrogen

The determination of nitrogen in soil was performed according to EN 13342 and DIN ISO 11261 standards. The samples were pre-treated by drying at 105 °C and milling using a mortar. A 0.5 g soil sample was digested with 8 mL of sulfuric acid (98%) and Kjeldahl Tablets (Buchi 11057980) in the KjelDigester K-449 (Buchi). After digestion, the samples were distilled in the KjelMaster system K-375/K-376 (Buchi) with 32 % NaOH and titrated with a 2 % boric acid solution. For quality control of the method, urea and blanks with ultrapure water were performed.

Soil respiration

Soil respiratory activity was determined by quantifying the production of carbon dioxide (CO_2). 25 g of sample of fresh soil were mixed with 0.01 g of ammonium nitrate and incubated at 28°C for 5 days . The released CO_2 was captured in 15 mL of 0.5 N sodium hydroxide, which was kept in a separate container, preventing direct contact with the soil. The trapped CO_2 was then titrated with 0.5 N hydrochloric acid in the presence of 2 % barium chloride. The amount of carbon dioxide was calculated using Equation S5.

$$\text{mg of } CO_2 = (B-V)\, NE \qquad \text{(S5)}$$

Where:

B: Volume (mL) of HCl used in the titration of the NaOH control.

V: Volume (mL) of HCl used in the NaOH titration of the studied soil.

N: Normality of hydrochloric acid.

E: Equivalent weight E=22 mg CO_2/mole HCl

Total Petroleum Hydrocarbons (TPH)

Total Petroleum Hydrocarbons (TPH) were identified using gas chromatography with a flame ionization detector (FID). The extraction process involved adding 10 mL of acetone to 10 g of fresh soil in a 40 mL VOA vial (Thermo Fisher) and stirring the mixture at 150 rpm on an orbital shaker for 30 minutes. Then, 10 mL of the TPH extraction solvent (hexane with extraction standard O-terphenyl) were added, and the mixture was stirred again under the same conditions. The sample was centrifuged at 1500 rpm for 3 minutes. Finally, 10 mL of deionized water was added to facilitate phase separation, and the organic phase was transferred to 2 mL amber glass vials. Negative control and positive spiked control (0.5 g/kg) were also prepared. The chromatographic conditions are detailed in Table S1. Certified diesel 2 (Absolute Standards, Inc 51006) was used for generating the primary calibration curve (5-50 mg/L). To prepare the spiked positive controls (e.g., 0.5 g/kg) and to represent the typical petroleum contamination, commercial diesel was utilized. This commercial diesel was adjusted to specific concentrations to serve as relevant internal controls for the extraction and analytical processes calibration. This method analyzes the TPH fraction ranging from C8 to C40.

Table S2. TPHs analysis GC conditions

Parameter	Value
Instrument model	Agilent Technologies 7890A GC System
Inlet	280 °C pulsed splitless
Injection volume	2 μL
Column	Fused Silica, Agilent 123.1632: DB-TPH (-10 °C – 320 °C (320°C): 30 m x 320 umx 0.25 μm)
Column temperature program	Initial temperature : 40°C (2 min). Ramp 1: 40°C/min to 150°C. Ramp 2: 30°C to 250°C. Ramp 3: 15°C to 310°C. Final temperature: 310°C (20 minutes).

Polycyclic Aromatic Hydrocarbons

For the analysis of PAHs, High Performance Liquid Chromatography with Fluorescence Detector (HPLC-FLD), was used as analytical technique. The extraction from soil samples followed and adapted QuEChERS-based method, involving extraction with acetonitrile-water, followed by salting-out parttioning with $MgSO_4$ and sodium acetate and a final clean-up step using Bond Elut Quechers (Agilent 5982-5158). This method is described in detail in [51].

Multielemental analysis

The dried soil samples were digested using a Microwave acid digestion system Ethos Up (Milestone, Sorisole, Italy) using Teflon vessels. First, a well homogenized portion of each sample (0.5 g), along with control samples: blank and spiked samples, were weighed in individual Teflon vessels. Next, 7 ml of bi-distilled HNO_3 were added to each sample using an acid dispenser. After manually homogenizing the sample, Table S2 shows the temperature ramps program used in the Microwave Digestion System.

Table S3. Multielemental soil digestion conditions

Initial Temperature (°C)	Final Temperature (°C)	Time (min)
Ambient	180	10
180	200	25
200	200	10

The digested and filtered samples were analyzed in a single quadrupole 7700x ICP-MS (Agilent Technologies, Santa Clara, California, US). Elements including Li, Be, B, Na, Mg, Al, Si, P, S, K, Ca, Ti, V, Cr, Mn, Fe, Co, Ni, Cu, Zn, As, Se, Sr, Mo, Ag, Cd, Sn, Sb, Ba, Hg, Tl were quantified using helium as collision gas in the octopole to minimize polyatomic interferences. Calibration curves were generated for group of elements: Heavy metals (1×10^{-4} - 6×10^{-2} mg/L), prevalent elements (0.2-450 mg/L) and anionics (0.2-600 mg/L), using mono-element ICP reference standards (Accustandard Inc.).

Soil microbial population assay

Table S4. Population assay media and dilutions for population counting

Population	Medium	Supplier/reference	Dilution*
Total bacteria	Nutrient agar	Difco (213000)	10^{-2} and 10^{-3}
Actinomycetes	ISP2	Difco (277010)	10^{-1} and 10^{-2}
Fungi	Rosa de Bengala	Merck (R1273)	10^{-4}, 10^{-6}, 10^{-8}
Phosphate solubilizing	Pikovskaya	Sigma-Aldrich (P1602)	10^{-1} and 10^{-2}
Yeast	YPD agar	Difco (242720)	10^{-1} and 10^{-2}
Siderophore producing	King B	Merck (60786)	10^{-1} and 10^{-2}

*Dilution spread method

Results

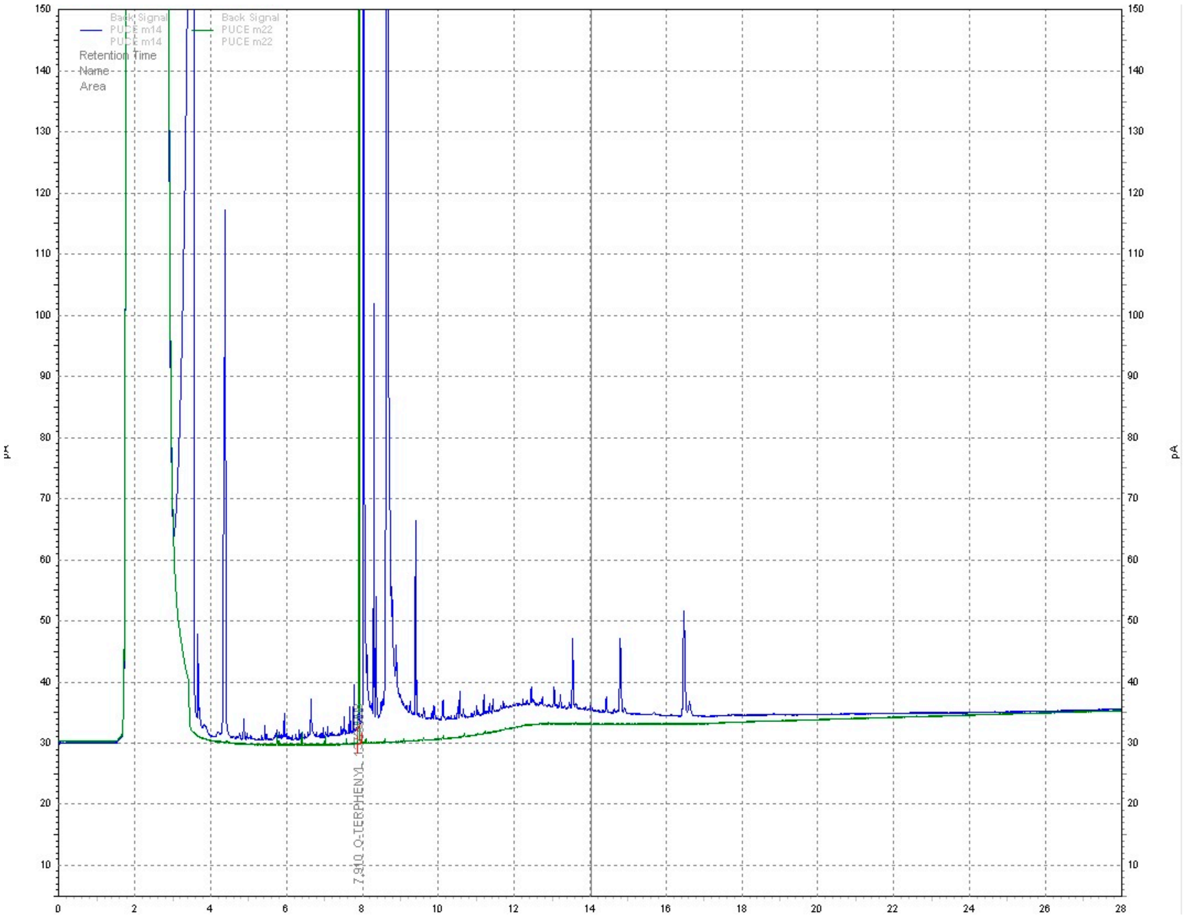

Figure S1. Chromatograms of one replicate of negative control (blue) and *Ganoderma cf.parvulum* (green) after the 60-day incubation period

Table S5. Rabbit food multielemental analysis

Atomic mass & Element	Result mg/kg
7 Li	7×10^{-5}
9 Be	3×10^{-6}
11 B	0.012
23 Na	1556
25 Mg	3484
27 Al	0.14
28 Si	1.4
31 P	7.8
34 S	2002
39 K	10501
44 Ca	7780
49 Ti	0.004

51 V	8×10^{-4}
52 Cr	0.002
55 Mn	0.081
56 Fe	0.19
59 Co	2×10^{-4}
60 Ni	0.002
63 Cu	0.008
66 Zn	0.068
75 As	1×10^{-4}
77 Se	4×10^{-4}
88 Sr	0.018
95 Mo	0.002
109 Ag	1×10^{-5}
111 Cd	5×10^{-5}
118 Sn	1×10^{-5}
123 Sb	2×10^{-5}
137 Ba	0.012
201 Hg	4×10^{-6}
208 Pb	1×10^{-4}

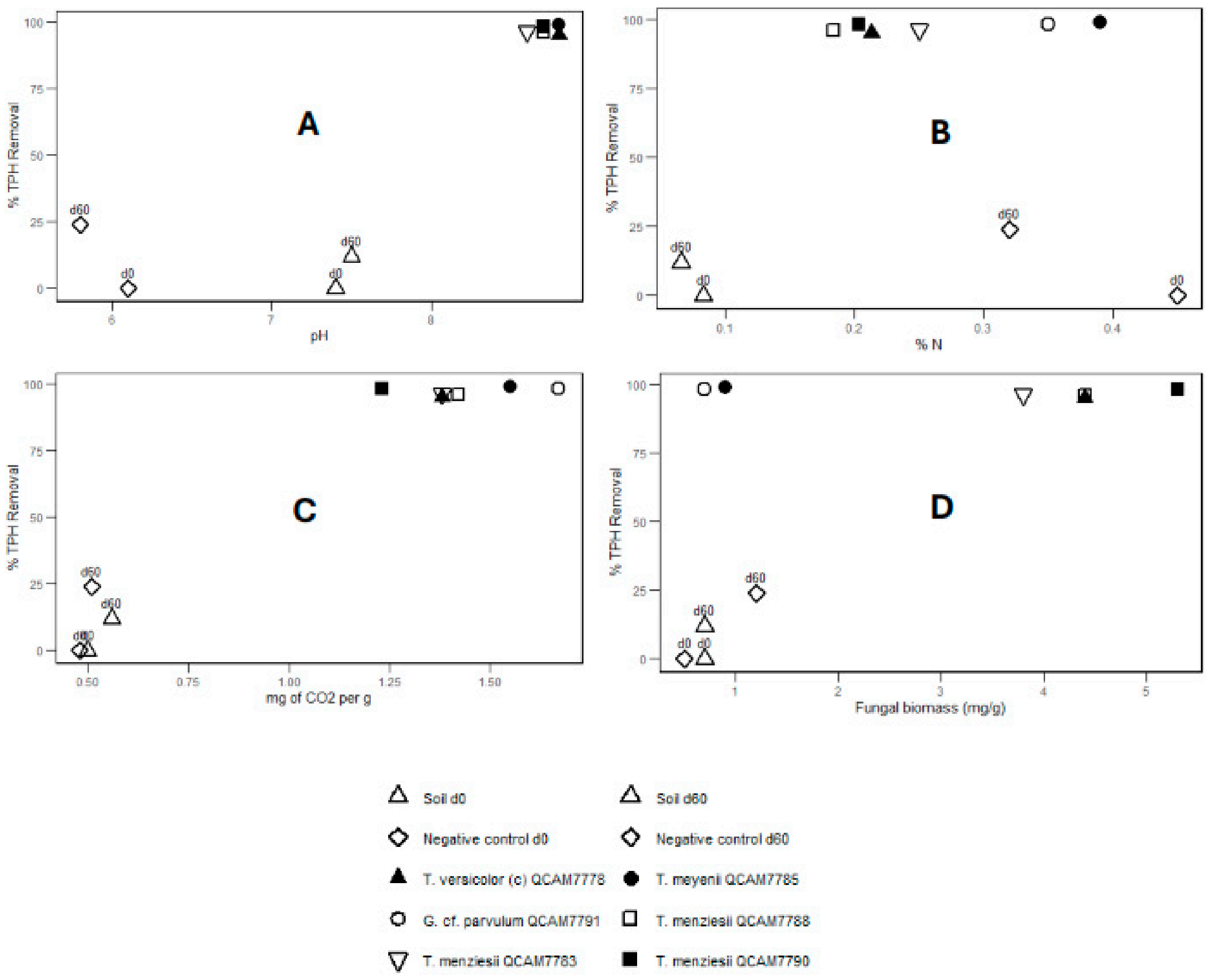

Figure S2. Relation between TPH removal yield with pH (A), % N (B), soil respiration (C) (mg of CO2/g_dry_soil) and Fungal biomass (mg of fungal biomass/g dry matter). TPH removal is calculated based on initial soil mass; other parameters are reported per unit of total dry matter, or per dry soil for controls without added substrate.
